# Severe Immune Thrombocytopenia Post-COVID-19: A Case Report

**DOI:** 10.7759/cureus.19544

**Published:** 2021-11-13

**Authors:** Adrian Davoodian, Chukwuemeka Umeh, Elli Novatcheva, Gabriela P Sassi, Hycienth Ahaneku, Ankur Kundu

**Affiliations:** 1 Internal Medicine, Hemet Global Medical Center, Hemet, USA; 2 Hematology and Oncology, Beaumont Health, Royal Oak, USA

**Keywords:** heparin induced thrombocytopenia, case report, itp, immune thrombocytopenia, covid-19

## Abstract

Immune thrombocytopenia, also called idiopathic thrombocytopenic purpura, is a common cause of thrombocytopenia after viral infections. Even in the second year of the coronavirus disease 2019 (COVID-19) pandemic, our body of knowledge regarding the diverse symptoms and complications of the virus continues to grow and evolve. We present a case of a 48-year-old African American male who came into the emergency department with severe left foot pain. A platelet count of 7x10^3^/mL was incidentally found on complete blood count (CBC) during the patient's initial workup. The patient had previously been hospitalized for COVID-19 five weeks prior. Further workup did not support any etiology of his thrombocytopenia. Therefore, we gave a presumed diagnosis of idiopathic thrombocytopenic purpura secondary to COVID-19. The patient was treated with corticosteroid resulting in improvement in his platelet count.

## Introduction

Since the World Health Organization declared the outbreak of coronavirus disease 2019 (COVID-19) a pandemic on March 11, 2020, researchers and clinicians have worked diligently to learn everything we can about the disease that has resulted in over 240 million cases and nearly five million deaths worldwide [1]. While most patients who become infected with COVID-19 recover without complications, some patients develop sequelae. One such complication of COVID-19 is immune thrombocytopenia (ITP) [2].

Immune thrombocytopenia is caused by autoantibodies against platelet antigens, resulting in thrombocytopenia. According to a review in the United States, the prevalence of ITP was approximately eight per 100,000 in children and 12 per 100,000 in adults [3], with the chronic disease being more common in adults. In the acute setting, ITP appears abruptly, often one to two weeks after a self-limited viral illness. The illness appears to trigger the development of autoantibodies through uncertain mechanisms. We present a case of a 48-year-old African American man who presented to the Emergency Department with gout, who was incidentally found to have severe thrombocytopenia, five weeks after being discharged from the hospital for COVID-19. This report underscores the importance of monitoring for COVID-19 sequelae and the potential to prevent severe bleeding events.

## Case presentation

A 48-year-old African American man with a medical history of COVID-19 pneumonia and obesity presented to the emergency department with left foot pain for the past three days. Initial workup returned a diagnosis of gout but also uncovered an incidental finding of a platelet count of 7x10^3^/mL. All other labs including white blood cell count and hemoglobin were normal. The patient stated he had been hospitalized for COVID-19 pneumonia five weeks prior but denied any lingering symptoms, had not noticed any bleeding and denied any home medications. Additionally, the patient denied any relevant family or social history. Vital signs were stable. The physical exam did not reveal any petechiae, purpura, palpable lymphadenopathy, or visceromegaly. 

Chart review showed that the patient's platelet count was 325x10^3^/ml when he was discharged from the hospital five weeks prior. Upon further workup for thrombocytopenia, a repeat complete blood count confirmed the initial finding, and microscopy did not reveal any clumping or abnormal platelet morphology. Coagulation studies were non-contributory. A viral panel, including HIV, hepatitis C virus (HCV), and Epstein-Barr Virus (EBV), was non-reactive and folate and B12 levels were within normal limits. Ultrasound of the spleen was unremarkable. Having ruled out other causes of thrombocytopenia, we made a diagnosis of secondary immune thrombocytopenia due to COVID-19.

The patient was admitted for observation and transfusion. The patient was given two units of platelets. The patient was also started on prednisone 40mg daily. He was discharged home on day two with a platelet count of 47x10^3^/ml. During follow-up as an outpatient two weeks later, complete blood count (CBC) revealed his thrombocyte count was 120x10^3^/ml.

## Discussion

Immune thrombocytopenia has been reported in COVID-19 patients. The mechanism of ITP is unclear but can be attributed to the underlying immune dysregulation in COVID-19, leading to the production of antiplatelet antibodies that destroy circulating platelets and megakaryocytes in the bone marrow [2,4]. The onset of ITP in COVID-19 patients occurs anytime during the disease [4]. However, 20% of ITP cases in a systematic review occurred three weeks after the onset of COVID-19 symptoms, with many occurring after clinic recovery [2]. For our patient, ITP was an incidental finding five weeks after COVID-19 infection and after complete clinical recovery. In prior studies, about 30% to 40% of COVID-19 ITP patients were asymptomatic [2,4]. When symptomatic, the commonest symptoms have been cutaneous bleeding, followed by epistaxis. However, severe bleeding, including intracranial hemorrhage and death resulting from hemorrhage, has been reported [2,4].

Diagnosis

ITP is a diagnosis of exclusion and is diagnosed in patients presenting with thrombocytopenia in which other possible causes of thrombocytopenia have been excluded and assessment of response to treatment [5,6]. The American Society of Hematology (ASH) recommends testing adults newly diagnosed with ITP for HIV, and hepatitis C [6]. Further investigations are required if there are other abnormalities in the blood count or smear other than thrombocytopenia. Bone marrow biopsies are usually not required in patients presenting with typical ITP [5,6]. However, in COVID-19 patients who received heparin prophylaxis, heparin-induced thrombocytopenia (HIT) should be excluded [2]. Patients with HIT usually have moderate thrombocytopenia with a platelet count above 50x10^3^/ml and rarely develop severe thrombocytopenia (<20x10^3^/ml) unless in cases of fulminant thrombosis or consumptive coagulopathy [7]. HIT can be ruled out using antiplatelet factor 4 (PF-4) or serotonin release assay test where available [3]. Furthermore, HIT can be excluded using the 4Ts score, which incorporates the severity of thrombocytopenia, the timing of thrombocytopenia to the onset of heparin use, complication of thrombosis, and exclusion of other causes of thrombocytopenia [8]. HIT can reliably be excluded with a low 4Ts score (≤3) because of its high negative predictive value [8].

Treatment

Treatment of ITP is dependent on the platelet count and whether the patient is bleeding. The goal of treatment is to treat or prevent significant bleeding in patients [9]. Patients with critical bleeding causing hemodynamic instability or those who bleed into critical anatomic sites like the brain or spine require platelet transfusion and intravenous immune globin (IVIG), and glucocorticoid [9]. Our patient received platelet transfusion to prevent bleeding because his platelet count was below 10x10^3^/ml; however, transfusion is not usually recommended in ITP patients who are not bleeding irrespective of the platelet count [9,10]. For patients with bleeding requiring blood transfusion or a fall in hemoglobin of 2g/dl or more but who are hemodynamically stable and who are not bleeding into critical anatomic sites, treatment is with IVIG, and corticosteroid used together for a rapid increase in platelet count [9].

In adults with a platelet count <30x10^3^/ml who are symptomatic or with minor mucocutaneous bleeding, the American Society of Hematology (ASH) recommends using corticosteroids as the first-line. However, IVIG may be used as first-line therapy if corticosteroids are contraindicated [5]. Conversely, observation without treatment is recommended in newly diagnosed ITP patients with platelets ≥30x10^3^/ml who are asymptomatic or with minor mucocutaneous bleeding [5]. If corticosteroids are used, the ASH guideline suggests either prednisone (0.5-2.0 mg/kg per day for ≤six weeks) or dexamethasone (40 mg per day for four days) for initial therapy. Similarly, if IVIG is used, the initial dose is 1 g/kg as a one-time dose, repeated if necessary [5]. Our patient was placed on prednisone 40mg daily for one week, and his platelet count two weeks later increased from 7x10^3^/ml to 120x10^3^/ml. 

## Conclusions

Thrombocytopenia predisposes patients to fatal bleeding events. Therefore, the presence of thrombocytopenia or bleeding in post-COVID patients should raise suspicions of ITP. Early recognition during routine follow-up could lead to prevention and better outcomes. We report this case to raise clinical awareness of potentially life-threatening ITP secondary to COVID-19.
